# Effective Management of Recurrent Conjunctival Intraepithelial Neoplasia Using Topical Mitomycin C 0.02%

**DOI:** 10.7759/cureus.110134

**Published:** 2026-06-02

**Authors:** Haya A Alnafisah, Mutlaq H Alshaibani, Mona I AlOmairini

**Affiliations:** 1 Ophthalmology Department, Prince Mohammed Bin Abdulaziz Hospital, Second Health Cluster, Riyadh, SAU; 2 Psychiatry Department, King Khalid University Hospital, Riyadh, SAU

**Keywords:** conjunctival intraepithelial neoplasia (cin), conjunctival neoplasia, mitomycin c (mmc), ocular surface neoplasia, recurrent cin, squamous cell carcinoma (scc), topical chemotherapy

## Abstract

Conjunctival intraepithelial neoplasia (CIN) is a premalignant lesion within the ocular surface squamous neoplasia (OSSN) spectrum that may recur after treatment and can progress to invasive squamous cell carcinoma (SCC) if left untreated. Management options include surgical excision, topical chemotherapeutic agents, immunotherapy, and radiotherapy in selected cases.

A 64-year-old Egyptian man presented with a recurrent conjunctival mass in the right eye associated with irritation and discomfort. He had undergone surgical excision of a similar lesion at the same site 10 years earlier, with histopathological confirmation of CIN. Examination revealed a 6 × 7 mm raised, vascularized, irregular gelatinous lesion on the nasal conjunctiva, extending from the caruncle to the limbus with approximately 2 mm of nasal corneal epithelial involvement. The recurrent lesion was treated without current repeat surgical excision using topical mitomycin C (MMC) 0.02% in a cyclic regimen. Marked clinical regression was observed after four weeks. A second course was administered for a small residual lesion, resulting in complete clinical regression with mild localized scarring. The treatment was well tolerated, with only mild transient ocular discomfort, conjunctival injection, and tearing. The patient was followed for more than one year after treatment completion, with no documented clinical recurrence.

This case suggests that topical MMC 0.02% may be an effective conservative treatment option for selected patients with recurrent CIN. Longer follow-up and larger studies are needed to confirm sustained efficacy, recurrence risk, and long-term safety.

## Introduction

Conjunctival intraepithelial neoplasia (CIN) is a premalignant epithelial lesion of the conjunctiva and cornea and represents the preinvasive end of the ocular surface squamous neoplasia (OSSN) spectrum. OSSN includes a range of lesions from mild epithelial dysplasia to carcinoma in situ and invasive squamous cell carcinoma (SCC) if left untreated [1-3]. CIN is clinically important because of its potential for local recurrence and progression to invasive disease [1].

Several risk factors have been associated with CIN, including ultraviolet radiation exposure, human papillomavirus (HPV) infection, human immunodeficiency virus (HIV) infection, immunosuppression, older age, and outdoor occupational exposure, particularly in regions with high ultraviolet exposure [1,2,4]. Clinically, CIN commonly presents as an elevated, leukoplakic, gelatinous, or vascularized lesion arising near the limbus. Variable pigmentation, feeder vessels, irregular borders, and corneal epithelial extension may also be observed [3].

Diagnosis is usually suspected clinically based on slit-lamp examination. Adjunctive imaging modalities, such as anterior segment optical coherence tomography (AS-OCT) and confocal microscopy, may help assess lesion extent, epithelial thickening, corneal involvement, and response to treatment [2,5-7]. However, histopathological examination remains the gold standard for definitive diagnosis, particularly when the clinical appearance is atypical or when invasive disease is suspected [2,5-7].

Management options include surgical excision with wide clear margins, cryotherapy, topical chemotherapeutic agents, immunotherapy, and radiotherapy in selected cases [5,6]. Surgical excision may be associated with recurrence, particularly when margins are involved or when diffuse ocular surface disease is present. Therefore, topical agents such as mitomycin C, 5-fluorouracil, and interferon alpha-2b are often used as primary or adjunctive therapies [5,6]. Mitomycin C is clinically useful because of its ability to treat diffuse or recurrent ocular surface disease and reduce the need for repeated surgical excision; however, careful monitoring is required because ocular surface toxicity may occur.

We present a case of recurrent CIN after previous surgical excision, successfully treated with topical mitomycin C 0.02% without current repeat surgical excision. This case highlights the potential role of low-dose topical mitomycin C as a conservative treatment option for recurrent CIN, particularly in cases where avoiding repeated surgery may be desirable.

## Case presentation

A 64-year-old male patient of Egyptian descent was referred for a comprehensive ophthalmic evaluation because of a recurrent conjunctival mass associated with irritation and discomfort in the right eye. He denied pain, photophobia, or significant visual disturbance. At presentation, the uncorrected visual acuity was 20/20 in both eyes.

**Figure 1:**
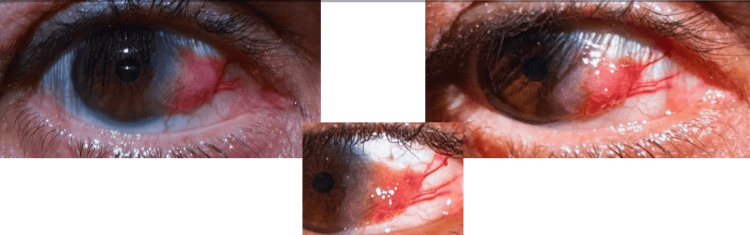
Pretreatment appearance of recurrent CIN (right eye) Pretreatment clinical photographs of the right eye showing a raised, vascularized, irregular gelatinous conjunctival mass on the nasal conjunctiva, extending from the caruncle to the limbus with approximately 2 mm of nasal corneal epithelial involvement. CIN: conjunctival intraepithelial neoplasia

On anterior segment examination, a raised, vascularized, irregular, and gelatinous mass measuring 6 x 7 mm was observed on the nasal conjunctiva of the right eye, extending from the caruncle to the limbus, with approximately 2 mm of nasal corneal epithelial extension (Figure 1). The posterior segment examination was unremarkable in both eyes. The patient reported that the lesion had gradually increased in size over five years.

The patient had a history of surgical excision of a similar lesion at the same site approximately 10 years earlier in Egypt, which was histopathologically diagnosed as conjunctival intraepithelial neoplasia (CIN). For the current lesion, no recent surgical excision or repeated biopsy was performed before treatment. The diagnosis was based on the previous histopathological diagnosis, recurrent clinical appearance, lesion morphology, and progression. The absence of repeat histopathological confirmation was considered a limitation and discussed with the patient.

Given the recurrent nature of the lesion and its clinical characteristics, conservative treatment with topical mitomycin C (MMC) 0.02% was initiated without current surgical excision. The regimen consisted of one drop four times daily for one week on, followed by one week off, then one week on, and one week off. Preservative-free lubricating eye drops were prescribed throughout the treatment course. The patient was reviewed weekly to assess lesion response and treatment tolerance. The treatment was generally well-tolerated, with only mild transient ocular discomfort, conjunctival injection, and tearing. No significant ocular surface complications were observed.

**Figure 2:**
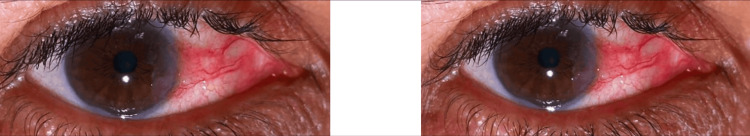
Clinical regression after four weeks of topical MMC 0.02% (right eye) Clinical photographs of the right eye obtained four weeks after initiating topical mitomycin C 0.02% showing marked regression of the nasal conjunctival lesion with reduced vascularity and residual localized conjunctival injection. MMC: mitomycin C

**Figure 3:**
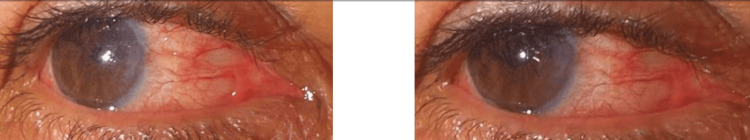
Near-complete regression after additional topical MMC 0.02% (right eye) Clinical photographs of the right eye obtained one week after the additional course of topical mitomycin C 0.02% showing near-complete regression of the previously noted nasal conjunctival lesion. MMC: mitomycin C

**Figure 4:**
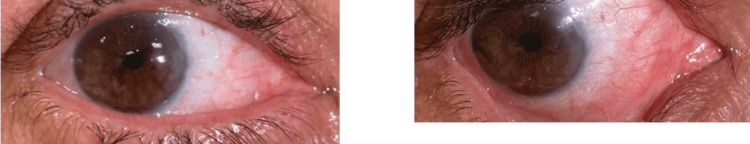
Complete clinical regression after topical MMC 0.02% (right eye) Posttreatment clinical photographs of the right eye showing complete clinical regression of the lesion, with no visible residual tumor and mild localized scarring at the site of the previous conjunctival epithelial lesion. MMC: mitomycin C

Four weeks after starting treatment, marked clinical regression of the lesion was noted (Figure 2). Because a small residual lesion remained, an additional course of topical MMC 0.02% was prescribed for two weeks using a modified cyclic regimen of one drop four times daily for four days on, three days off, four days on, and three days off. At the one-week follow-up visit after the additional course, the lesion had almost completely resolved (Figure 3). Upon completion of treatment, complete clinical regression was documented, with residual localized scarring at the site of the previous epithelial lesion (Figure 4).

The patient continued regular follow-up after treatment completion for more than one year. During the documented follow-up period, the lesion remained clinically resolved with no evidence of recurrence. Long-term surveillance was recommended because of the known risk of recurrence in CIN.

## Discussion

Conjunctival intraepithelial neoplasia (CIN) represents the preinvasive end of the ocular surface squamous neoplasia (OSSN) spectrum, involving the conjunctiva and cornea. It includes varying degrees of epithelial dysplasia and carcinoma in situ, while invasive squamous cell carcinoma represents progression beyond the epithelial basement membrane [1,8]. Accurate classification is important because CIN may recur and, in some cases, progress to invasive disease if inadequately treated.

Histopathological examination remains the gold standard for definitive diagnosis. However, adjunctive diagnostic tools, including impression cytology, vital dye staining, anterior segment optical coherence tomography (AS-OCT), and confocal microscopy, may support clinical assessment and help evaluate lesion extent and response to treatment [1,9]. AS-OCT can demonstrate features such as epithelial thickening and a clear transition between abnormal and normal epithelium, which may be helpful in differentiating CIN from surrounding healthy tissue [1,9]. In the present case, the recurrent lesion was managed clinically based on the patient’s previous histopathological diagnosis of CIN, typical recurrent appearance, gradual progression, and response to treatment. Repeat biopsy and AS-OCT imaging were not performed for the recurrent lesion, which represents an important limitation.

The etiology of CIN is multifactorial. Several studies have linked long-term ultraviolet radiation exposure to increased risk, particularly among individuals living in regions with high ultraviolet exposure [1,10]. Human papillomavirus (HPV), human immunodeficiency virus (HIV), and immunosuppression have also been associated with CIN, suggesting a potential role for viral oncogenesis and impaired immune surveillance [11]. Clinically, patients may present with a unilateral elevated lesion, conjunctival redness, irritation, foreign body sensation, tearing, or visual disturbance when the lesion extends toward the visual axis [12]. In this patient, the lesion was unilateral, raised, vascularized, irregular, and gelatinous, located on the nasal conjunctiva with corneal epithelial extension, consistent with common clinical descriptions of CIN.

Recurrence after treatment has been widely reported, with recurrence rates in CIN ranging from approximately 20% to 40% in some studies [5]. Recurrence may occur because of difficulty in clinically delineating lesion borders, microscopic residual disease, multifocal dysplastic changes, or involvement of the limbal stem cell region [5]. Although surgical excision with clear margins is an established treatment option, recurrence may still occur even after apparently complete excision. Therefore, topical therapies may be useful, particularly in recurrent, diffuse, or limbal lesions where repeated surgery may increase ocular surface morbidity.

Treatment strategies aim to eradicate neoplastic tissue while preserving ocular surface integrity. Topical agents, including mitomycin C (MMC), 5-fluorouracil, and interferon alpha-2b, have been used either as primary therapy or as adjunctive treatment after surgical excision [2,5,6,13]. MMC is an alkylating antimetabolite that inhibits DNA synthesis and can treat the entire ocular surface, including clinically subtle or multifocal disease. Higher concentrations, such as MMC 0.04%, have been reported in the literature with favorable clinical responses; however, MMC may also be associated with ocular surface toxicity. Dudney and Malecha reported temporary conjunctival hyperemia and an epithelial defect after topical MMC treatment in a patient with recurrent CIN, with subsequent permanent limbal stem cell deficiency [14]. In the present case, topical MMC 0.02% was used without current repeat surgical excision and resulted in complete clinical regression of the recurrent lesion, with only mild transient ocular discomfort, conjunctival injection, and tearing.

This case supports the potential role of low-dose topical MMC 0.02% as a conservative treatment option for recurrent CIN. The lesion showed marked regression after the initial treatment cycle and complete clinical regression after an additional course. The patient was monitored for more than one year after treatment completion, with no documented clinical recurrence during the follow-up period. Nevertheless, continued long-term surveillance remains necessary because CIN can recur even after apparent clinical resolution.

This report has several limitations. Repeat histopathological confirmation was not performed for the recurrent lesion before treatment, and microscopic residual disease cannot be completely excluded based on clinical appearance alone. Adjunctive AS-OCT imaging and objective epithelial thickness measurements were not available. In addition, although the patient had more than one year of documented follow-up without recurrence, longer follow-up would further strengthen conclusions regarding sustained remission and long-term safety. As this is a single case report, the findings should be interpreted cautiously and cannot establish the overall efficacy or safety of MMC 0.02% for all patients with recurrent CIN. Larger studies with longer follow-up are needed to better define optimal dosing, recurrence risk, comparative efficacy, and adverse-effect profiles.

## Conclusions

This case suggests that topical mitomycin C (MMC) 0.02% may be an effective conservative treatment option for recurrent conjunctival intraepithelial neoplasia (CIN) in selected patients. In this patient, complete clinical regression was achieved without current repeat surgical excision and with only mild transient adverse effects. The lesion remained clinically resolved with no evidence of recurrence during more than one year of documented follow-up. However, longer follow-up and larger studies are needed to confirm sustained efficacy, recurrence risk, and long-term safety.
